# Geriatric screening tools are of limited value to predict decline in functional status and quality of life: results of a cohort study

**DOI:** 10.1186/s12875-015-0241-x

**Published:** 2015-03-03

**Authors:** Laura Deckx, Marjan van den Akker, Liesbeth Daniels, Eric T De Jonge, Paul Bulens, Vivianne CG Tjan-Heijnen, Doris L van Abbema, Frank Buntinx

**Affiliations:** 1 Department of General Practice, KU Leuven, Kapucijnenvoer 33, bus 7001, 3000 Leuven, Belgium; 2 Department of Family Medicine, CAPHRI School for Public Health and Primary Care, Maastricht University Medical Centre, P.O. Box 616, 6200 MD, Maastricht, The Netherlands; 3 Department of Gynaecology, Ziekenhuis Oost-Limburg, Schiepse Bos 6, 3600 Genk, Belgium; 4 Limburgs Oncologisch Centrum, Stadsomvaart 11, 3500 Hasselt, Belgium; 5 Department of Medical Oncology, GROW School for Oncology and Developmental Biology, Maastricht University Medical Centre, PO Box 5800, 6202 AZ, Maastricht, The Netherlands

**Keywords:** Neoplasms, Functional status, Quality of life, Geriatric oncology, Longitudinal study

## Abstract

**Background:**

Geriatric screening tools are increasingly implemented in daily practice, especially in the oncology setting, but also in primary care in some countries such as the Netherlands. Nonetheless, validation of these tools regarding their ability to predict relevant outcomes is lacking. In this study we evaluate if geriatric screening tools predict decline in functional status and quality of life after one year, in a population of older cancer patients and an older primary care population without cancer with a life expectancy of at least six months.

**Methods:**

Older cancer patients and a general older primary care population without a history of cancer (≥70 years) were included in an on-going prospective cohort study. Data were collected at baseline and after one-year follow-up. Functional decline was based on the Katz Index and Lawton IADL-scale and was defined as deterioration on one or more domains. Decline in quality of life was measured using the global health related subscale of the EORTC QLQ-C30, and was defined as a decline ≥10 points. The selected geriatric screening tools were the abbreviated Comprehensive Geriatric Assessment, Groningen Frailty Indicator, Vulnerable Elders Survey-13, and G8. We calculated sensitivity, specificity, predictive values, and odds ratios to assess if normal versus abnormal scores predict functional decline and decline in quality of life.

**Results:**

One-year follow-up data were available for 134 older cancer patients and 220 persons without cancer. Abnormal scores of all screening tools were significantly associated with functional decline. However, this was only true for older persons without cancer, and only in univariate analyses. For functional decline, sensitivity ranged from 54% to 71% and specificity from 33% to 66%. For decline in quality of life, sensitivity ranged from 40% to 67% and specificity from 37% to 54%.

**Conclusion:**

In older persons with a relatively good prognosis, geriatric screening tools are of limited use in identifying persons at risk for decline in functional status or quality of life after one year. Hence, a geriatric screening tool cannot be relied on in isolation, but they do provide very valuable information and may prompt physicians to also consider different aspects of functioning.

**Electronic supplementary material:**

The online version of this article (doi:10.1186/s12875-015-0241-x) contains supplementary material, which is available to authorized users.

## Background

Given the current demographic changes, and because cancer is mainly a disease of older persons, the number of older cancer patients is rising [1]. Currently, almost half of cancer survivors are aged 70 years or older [2]. As such, primary, as well as secondary care providers will be increasingly confronted with this growing group of patients.

Older cancer patients form a very heterogeneous group of patients. Among persons aged 70 years and above, there is a substantial variety in levels of fitness, comorbidity, and physiological reserve. Hence, treatment decisions related to cancer (oncologist) or general health (primary care) should not be based on chronological age alone. Therefore, clinicians have been searching for ways to obtain a better view on the global health status and reserve capacities of older cancer patients [3]. Currently, one’s global health status and reserve capacities are best estimated by a geriatric assessment, which has been defined as a multidisciplinary evaluation of an older individual’s functional status, comorbidity, cognition, psychological status, social support, nutritional status and review of the patient’s medications [4,5]. Reasons for a geriatric assessment may vary, depending on the treatment decisions that have to be made. In oncology, the rationale behind a geriatric assessment is to assess if, for a given patient, anti-cancer treatment would do more good than harm [3,6]. Oncologists want to answer the following questions: what is the life expectancy of this patient; will the tumour influence overall survival and Quality of Life (QoL), or are other competing causes of death/disability of more importance? For generalists, such as general practitioners (GPs), the rationale behind a geriatric assessment is to assess how illness impacts functioning and what the social and medical needs are in order to develop a plan for treatment and follow-up, to manage the problems that were identified, and to prevent dependency [7].

Unfortunately, a geriatric assessment is very time consuming. Therefore, a two-step approach using geriatric screening tools has been suggested [8]. Numerous geriatric screening tools have been developed and especially in the oncology setting, these are increasingly implemented in daily practice. Also in some countries such as the Netherlands, geriatric screening tools are also implemented in primary care (see for example [9]). The majority of studies have focused on assessing their ability to identify patients who might benefit from a full geriatric assessment [10]. A recent update of the SIOG (International Society of Geriatric Oncology) recommendations showed that screening tools are indeed useful in order to identify those patients in need of a full geriatric assessment [11]. However, the predictive value of these screening tools remains open to debate. In older cancer patients, only a handful of studies focussed on their ability to predict mortality and treatment toxicity [12], only one study has focused on their ability to predict functional decline [13], and to our knowledge there are no studies that evaluated decline in QoL, although it is increasingly recognised that functional status and QoL are relevant outcomes, especially for older patients. As shown by others, adequate validation of these tools with respect to their value to predict functional decline and decline in QoL is lacking [10,13]. In primary care, the prediction of functional decline has received slightly more attention compared to older cancer patients but nonetheless the predictive abilities of several geriatric screening tools remains open to debate.

Hence, the aim of this study is to evaluate the value of four geriatric screening tools to predict functional decline and decline in QoL in a population of older cancer patients with a considerable life expectancy (>6 months), as especially in these patients functional decline and decline in QoL might be relevant outcomes. The timeframe of this study is a one-year period. This is a very relevant time frame for primary care as for cancer patients, this often corresponds with the transition from secondary to primary care, i.e. the time when the general practitioner becomes the first contact person again. Previous studies have shown that the role of general practitioners in cancer (after) care will become more prominent due to the increasing numbers of older cancer patients, and the shift from inpatient to ambulatory care [14-16].

Furthermore, we also aim to evaluate the predictive value of these tools in a general older primary care population without a history of cancer, because functional decline and decline in QoL are also relevant outcomes in this population and geriatric screening tools are also increasingly being implemented in primary care [17,18].

## Methods

### Design and study population

The data for this study were collected as part of the KLIMOP-study (Dutch acronym for “Kanker bij Limburgse en Vlaams-Brabantse Ouderen Project”) [19]. KLIMOP is an on-going observational cohort study of older cancer patients and a general older primary care population without a history of cancer (except non-melanoma of the skin), aged 70 years and above. The focus of this study is the long-term wellbeing of older cancer patients.

For the current study we selected only cancer patients with a new diagnosis of breast or colorectal cancer, and cancer stage I – III. We made this choice in order to have a more homogeneous sample for the analysis. They were recruited through seven hospitals in Belgium and the Netherlands, within three months after cancer diagnosis. Older patients without cancer were recruited through general practices in Belgium and the Netherlands in the same regions as the cancer patients. General practitioners asked all consecutive eligible patients (70 years and above, no history of cancer) to participate until 20 patients per general practitioner had agreed to participate.

Exclusion criteria for older cancer patients as well as persons without cancer were the inability to speak Dutch, a formal diagnosis of dementia, and an estimated life expectancy less than six months. For this study we present data of all patients included in the study between June 2010 and August 2012, who also participated in the data collection after one year.

### Data collection

Methods of data collection and management were identical in both groups. Data were collected through personal interviews or self-administered questionnaires, at baseline and at one-year follow-up. Data collection included socio-demographic information, medical information (number of medicines, type of cancer, cancer stage, and anti-cancer treatments received in the first year after diagnosis), functional status, a QoL questionnaire, and four geriatric screening tools. For the questionnaires such as functional status and QoL, people are asked to indicate for each statement the extent to which they apply their situation of the past week.

### Dependent variables

#### Decline in functional status

Functional status was measured by Activities of Daily Living (ADL) using the Katz Index [20] and by Instrumental Activities of Daily Living (IADL) using the Lawton IADL-scale [21]. The Katz Index consists of six items (bathing, dressing, toileting, transferring, continence and feeding); each item was scored as dependent versus independent. The total sum score ranges from 0 (dependent) to 6 (independent). The Lawton IADL-scale consists of eight items in women (ability to use the telephone, shopping, cooking, housekeeping, doing laundry, taking own medication, making transports, and ability to handle finances), and only five items in men, because cooking, housekeeping and doing laundry are not always applicable to men. Each item was scored as dependent versus independent. The total sum score ranges from 0 (dependent) to 8 (independent) for women, and from 0 (dependent) to 5 (independent) for men. Functional impairment was defined as dependency on at least one domain of ADL (score <6) or IADL (a score <8 from women or <5 for men). Functional decline was defined as deterioration on at least one domain of ADL or IADL compared to baseline (decline ≥1 point).

#### Decline in quality of life (QoL)

QoL was measured with the European Organization for Research and Treatment of Cancer Quality of Life Questionnaire C30 version 3 (EORTC QLQ-C30) [22]. The questionnaire consists of 30 questions, which correspond to five functional scales, three symptom scales, six single-item scales, and a global health related QoL scale. In this study, we only report the global health related QoL, from this point forward indicated as QoL. QoL is assessed by two Likert scales, ranging from one to seven, that measure a person’s perception on the health status and QoL of the past week. Based on the EORTC QLQ-C30 manual, QoL was recoded in a score ranging from 0 – 100, with higher scores representing better QoL [23]. The QoL subscale has high internal consistency with Cronbach’s alpha ranging between 0.82 and 0.89 [22]. In this study Cronbach’s alpha was 0.78 for older cancer patients and 0.76 for older persons without cancer. Decline in QoL was defined as a difference ≥10 points, which indicates a clinically important change [24].

### Independent variables

**Figure 1:**
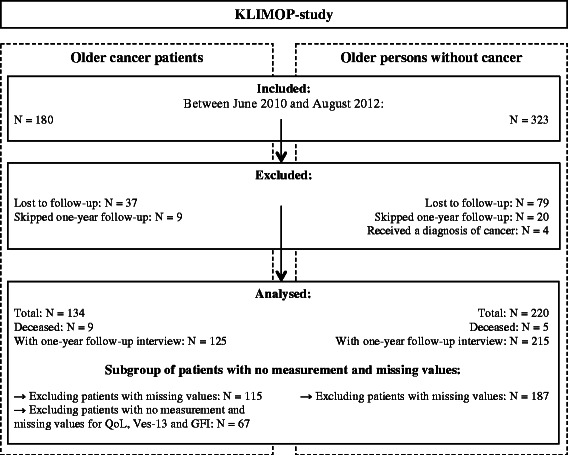
**Flow-chart: population of the KLIMOP-study.**

The geriatric screening tools included the abbreviated Comprehensive Geriatric Assessment (aCGA), the G8, the Groningen Frailty Indicator (GFI), and the Vulnerable Elders Survey-13 (VES-13). In one hospital, data collection was integrated in a routine geriatric assessment, and therefore, data collection was slightly different in this hospital; here QoL, GFI and VES-13 were not assessed. Hence, for 35 older cancer patients no data were available for these measurements. We always indicate the number of patients included in the analyses and a detailed flowchart of the patient population is provided in Figure 1.

#### The abbreviated comprehensive geriatric assessment (aCGA)

The aCGA was developed as a screening tool to identify older cancer patients who might benefit from an entire geriatric assessment. The aCGA was based on a chart review of more than 500 cancer patients [25,26]. The selection of items was based on psychometric criteria; those items within each scale that showed the highest item-to-total correlation were selected. As such, the aCGA includes four items from the 15-item Geriatric Depression Scale, three items from ADL, four items from IADL, and four items from the Mini Mental State Examination. A cut-off value was identified for each domain, indicating whether a more elaborate assessment was needed for that domain: ≥1 for the selected items of ADL and IADL; ≤6 for the items of the Mini Mental State Examination; and ≥2 for the items of the 15-item Geriatric Depression Scale [27]. In this study, we operationalized abnormal scores on the aCGA as impairment on at least one domain; this is in agreement with previous studies [27,28].

#### The G8

The G8 was designed to identify older cancer patients who might benefit from an entire geriatric assessment [29]. The G8 consists of eight questions relating to age, nutritional status, weight loss, body mass index, mobility, psychological status, number of medications, and self-perception of health [29]. The maximum score is 17 points. An abnormal score on the G8 was defined by the original cut-off value of ≤14.

#### The Groningen Frailty Indicator (GFI)

The GFI was developed as a short screening tool, aiming to identify patients that have diminished reserves in one or more of the fundamental domains of functioning and was originally tested in a sample of hospital inpatients, nursing home residents, and community-dwelling elderly [30]. It has been widely used in older cancer patients [10], as well as older primary care populations [18]. The GFI assesses mobility, assistance needed with toileting and shopping, physical fitness, poor hearing and vision, medicine use, weight loss, and complaints about memory, feelings of isolation, depression, and anxiety [30]. It consists of 15 questions with a maximum score of 15 points. Abnormal score for the GFI was defined by the original cut-off value of ≥4.

#### The Vulnerable Elders Survey-13 (VES-13)

The VES-13 was originally developed for identifying community-dwelling vulnerable older people at increased risk of death or functional decline [31], but has also been frequently used in older cancer patients [10]. The VES-13 is a simple 13-item screening tool including questions about older peoples’ ability to perform six physical and five functional activities, their self-rated health, and their age [31]. The maximum score is 10 points; an abnormal score was defined by the original cut-off value of ≥3.

### Ethics

The Ethical Review Board of KU Leuven and UZ Leuven (S52097 – ML6279) and the Maastricht University Medical Centre (NL31414.068.10) approved the study protocol. The study was conducted in compliance with Good Clinical Practice guidelines Procedures, the principles of the Declaration of Helsinki (version October 2008) and the Belgian (law of 7 may 2004 concerning clinical trials with humans) and Dutch (Medical Research Involving Human Subjects Act and Personal Data Protection Act) laws. All patients signed informed consent.

### Analysis

Sociodemographic and clinical characteristics of the study population are presented as the mean and standard deviation (SD) for continuous variables and as numbers and proportions for categorical variables. Comparisons between different groups (older cancer patients versus older persons without cancer) were performed using t-test for continuous data and the chi square test for categorical data. Furthermore, we compared persons that were included and excluded from analyses. Throughout all analyses, we handled older cancer patients and persons without cancer as separate groups, and a *p* value < 0.05 was considered to be statistically significant.

We calculated sensitivity, specificity, predictive values, and area under the receiver operating curve (AUC) with their 95% confidence intervals (CI) to assess if normal versus abnormal scores on the geriatric screening tools are able to predict functional decline and decline in quality of life after one year. Furthermore, Odds Ratios (OR) and corresponding 95% Confidence Intervals (CI) were calculated to compare the risk for decline in functional status and QoL after one-year follow-up in persons with normal versus abnormal scores on the geriatric screening tools. Univariate and multivariate logistic regression models were used. In multivariate models age, sex, and number of medicines were included as covariables. For cancer patients also cancer stage, cancer type, and treatment type were included as covariables.

As a sensitivity analysis, we repeated the analyses, including participants who died during one-year follow-up. In this case, mortality, defined as death within one year after baseline evaluation, was included as functional decline or decline in QoL. For some patients data for the geriatric screening tools were missing. Therefore we performed a sensitivity analysis to assess the influence of these missing values, making a worst- and best-case scenario by imputing missing values either as a normal or abnormal score. Furthermore, the proportion of women in the group of cancer patients was higher as compared to the primary care population without cancer due to the inclusion of breast cancer patients. As a sensitivity analysis, we repeated the analyses for women separately. We also aimed to assess the influence of our operationalization of functional decline. Therefore, we repeated the analyses for decline on ADL and IADL separately; decline on ADL was defined as deterioration on one domain of the ADL scale (decrease ≥1 point) and decline on IADL was defined as deterioration on one domain of the IADL scale (decrease of ≥1 point).

Statistical analyses were performed using the STATA statistical software package version 11 (StataCorp LP, College Station, TX).

## Results

### Population

In total 503 participants were recruited. For 354 (70%) participants follow-up data were available, baseline characteristic are presented in Table 1. All patients were Caucasian. The mean age of cancer patients was comparable to the mean age of persons without cancer (77.10 and 78.13 respectively). The proportion of women was higher in the cancer group compared to older persons without cancer, due to the recruitment of breast cancer patients (*p* < 0.01). Between 42% and 64% of older cancer patients had abnormal scores on the screening tools, and between 45% and 56% of older persons without cancer had abnormal scores on the screening tools (see Table 1). The prevalence of abnormal scores was comparable in persons with and without cancer for all screening tools except for the aCGA (*p* < 0.04). Seventy-two percent of older cancer patients and 65% of persons without cancer were functionally impaired at baseline (*p* = 0.12). The baseline mean QoL was significantly lower for older cancer patients compared to older persons without cancer (*p* < 0.01).

**Table 1:** **Baseline population characteristics**

	Older cancer patients		Older persons without cancer		
	N = 134		N = 220		
	N	%	N	%	*p*value
**Age: mean (SD)**	77.10	(5.11)	78.13	(5.47)	0.08
**Gender**					0.001
Male	28	21%	82	37%	
Female	106	79%	138	63%	
**Living conditions**					0.17
Alone	44	33%	72	33%	
With partner	76	57%	137	62%	
With friends/family	6	4%	7	3%	
Institutionalised	8	6%	4	2%	
**Cancer site**					
Breast	93	69%			
Colorectal	41	31%			
**Cancer treatment**					
Surgery	125	93%			
Chemotherapy	39	29%			
Radiotherapy	70	52%			
Hormonal therapy	53	40%			
Targeted therapy	4	3%			
Missing	3	2%			
**Cancer Stage**					
I	22	16%			
II	72	54%			
III	18	13%			
Missing	22	16%			
**Geriatric screening tools**					
aCGA: Abnormal score	86	64%	123	56%	0.04
Missing	12		12		
G8: Abnormal score	84	63%	119	54%	0.10
Missing	3		4		
GFI: Abnormal score	42	42%	98	45%	0.58
Missing	17		14		
Not measured^a^	35		/		
VES-13: Abnormal score	48	48%	109	50%	0.92
Missing	5		4		
Not measured^a^	35		/		
**Functional impairment** ^**b**^	97	72%	141	65%	0.12
Missing	0		0		
ADL impairment	69	51%	87	41%	0.06
IADL impairment	75	56%	113	51%	0.40
Functional decline	73	58%	93	43%	0.01
**QoL: mean (SD)** ^**a**^	69.93	(19.30)	75.91	(17.84)	0.01
Decline in QoL	15	23%	50	30%	0.48
Missing	7		1		
Not measured^a^	35		/		

*Abbreviations:aCGA* abbreviated Comprehensive Geriatric Assessment, *GFI* Groningen Frailty Indicator, *VES-13* Vulnerable Elders Survey-13, *ADL* activities of daily living, *IADL* instrumental activities of daily living, *QoL* Quality of Life.

^a^In one hospital, data collection was integrated in a routine geriatric assessment, therefore QoL, GFI and VES-13 were not assessed in the 35 older cancer patients recruited in this hospital. Decline in QoL was defined as a difference ≥10 points.

^b^Functional impairment was defined as impairment on at least one domain of ADL or IADL. Decline in functional status was defined as deterioration on at least one domain of ADL or IADL compared to baseline.

### Follow-up

During the one-year follow-up period, 9 older cancer patients and 5 persons without cancer died (*p* < 0.01). Of the 503 participants, 116 (23%) were lost to follow-up and 29 (6%) patients skipped data collection at one-year follow-up (see Figure 1). Patients who skipped data collection were still willing to participate in future follow-up visits (e.g. at three years follow-up). Reasons for skipping the interview at one-year follow-up were personal problems (e.g. loss of a spouse), health problems (e.g. cancer recurrence), and other (e.g. too busy). The percentage of older cancer and persons without cancer that were lost to follow-up or skipped data collection at one-year follow-up was comparable (*p* = 0.93). Furthermore, cancer patients with one-year follow-up data were similar to those without follow-up data with respect to age, sex, living conditions, cancer type, and functional status at baseline. Only for cancer stage, the proportion of excluded cancer patients with stage II cancer was lower, and the proportion with stage III cancer was higher, as opposed to included cancer patients (*p* < 0.04). Also for person without cancer, baseline characteristics were comparable for those with and without one-year follow-up (see Additional file 1: Table S1).

### Predictive value of geriatric screening tools

#### Functional decline

After one-year follow-up, 58% of older cancer patients experienced functional decline, compared to 43% of persons without cancer (*p* < 0.01). Abnormal scores at baseline for all four geriatric screening tools were significantly associated with functional decline after one year, but only among persons without cancer, and only in univariate analyses. In multivariate analyses, only abnormal score on the VES-13 was significantly associated with functional decline in older persons without cancer (OR_VES-13_ 2.83, 95% CI: 1.35 – 5.95). The predictive properties of all screening tools were poor with sensitivity ranging between 54% and 71% for functional decline (see Table 2). Specificity ranged between 33% and 66%. The positive predictive value ranged between 49% and 65% and the negative predictive value ranged between 43% and 72%. The AUC ranged between 0.50 and 0.64, hence, also the ability of the selected geriatric screening tools to differentiate between persons that will or will not experience functional decline is poor.

**Table 2:** **Diagnostic accuracy of geriatric screening tools to predict decline in functional status and QoL**

			Sensitivity		Specificity		Positive predictive value		Negative predictive value		Area under the curve		Univariate logistic regression		Multivariate logistic regression^c^	
		N total	Se	(95% CI)	Sp	(95% CI)	PPV	(95% CI)	NPV	(95% CI)	AUC	(95% CI)	OR	(95% CI)	OR	(95% CI)
**Functional decline** ^**a**^																
**aCGA**	Cancer patients	115	71%	(59% - 82%)	33%	(20% - 48%)	59%	(47% - 70%)	46%	(29% - 63%)	0.52	(0.43 - 0.61)	1.20	(0.54 - 2.67)	0.61	(0.23 - 1.64)
	Persons without cancer	187	67%	(55% - 78%)	52%	(43% - 62%)	49%	(39% - 59%)	70%	(59% - 80%)	0.60	(0.53 - 0.67)	**2.23**	**(1.22 - 4.09)**	1.87	(0.93 - 3.78)
**G8**	Cancer patients	115	64%	(51% - 75%)	37%	(23% - 52%)	58%	(45% - 69%)	43%	(28% - 59%)	0.50	(0.41 - 0.59)	1.02	(0.47 - 2.19)	0.67	(0.28 - 1.65)
	Persons without cancer	187	65%	(53% - 75%)	57%	(47% - 66%)	51%	(40% - 61%)	70%	(59% - 79%)	0.61	(0.54 - 0.68)	**2.38**	**(1.31 - 4.35)**	1.80	(0.90 - 3.60)
**GFI**	Cancer patients	67	54%	(37% - 71%)	59%	(41% - 76%)	59%	(41% - 76%)	54%	(37% - 71%)	0.57	(0.45 - 0.69)	1.74	(0.66 - 4.58)	1.23	(0.35 - 4.35)
	Persons without cancer	187	58%	(46% - 69%)	63%	(53% - 72%)	52%	(41% - 63%)	69%	(59% - 78%)	0.61	(0.53 - 0.68)	**2.35**	**(1.29 - 4.26)**	1.83	(0.92 - 3.65)
**VES-13**	Cancer patients	67	57%	(39% - 74%)	66%	(47% - 81%)	65%	(45% - 81%)	58%	(41% - 75%)	0.61	(0.50 - 0.73)	2.55	(0.95 - 6.85)	2.58	(0.59 - 11.22)
	Persons without cancer	187	62%	(50% - 73%)	66%	(56% - 75%)	55%	(44% - 66%)	72%	(62% - 80%)	0.64	(0.57 - 0.71)	**3.11**	**(1.70 - 5.71)**	**2.83**	**(1.35 - 5.95)**
**Decline in Qol** ^**b**^																
**aCGA**	Cancer patients	67	47%	(21% - 73%)	37%	(24% - 51%)	18%	(7% - 33%)	70%	(50% - 86%)	0.42	(0.27 - 0.56)	0.50	(0.16 - 1.61)	0.44	(0.10 - 1.91)
	Persons without cancer	187	56%	(41% - 70%)	45%	(36% - 53%)	27%	(19% - 37%)	74%	(63% - 83%)	0.50	(0.42 - 0.58)	1.02	(0.53 - 1.96)	1.14	(0.55 - 2.39)
**G8**	Cancer patients	67	67%	(38% - 88%)	39%	(25% - 53%)	24%	(12% - 40%)	80%	(59% - 93%)	0.53	(0.39 - 0.67)	1.25	(0.37 - 4.19)	1.47	(0.36 - 6.05)
	Persons without cancer	187	40%	(26% - 55%)	44%	(35% - 53%)	21%	(13% - 30%)	67%	(56% - 76%)	0.42	(0.34 - 0.50)	0.52	(0.27 - 1.00)	0.48	(0.22 - 1.03)
**GFI**	Cancer patients	67	40%	(16% - 68%)	50%	(36% - 64%)	19%	(7% - 36%)	74%	(57% - 88%)	0.45	(0.30 - 0.60)	0.67	(0.21 - 2.14)	0.50	(0.11 - 2.20)
	Persons without cancer	187	42%	(28% - 57%)	53%	(45% - 62%)	25%	(16% - 35%)	72%	(62% - 80%)	0.48	(0.40 - 0.56)	0.83	(0.43 - 1.59)	0.89	(0.42 - 1.88)
**VES-13**	Cancer patients	67	47%	(21% - 73%)	54%	(40% - 68%)	23%	(10% - 41%)	78%	(61% - 90%)	0.50	(0.36 - 0.65)	1.02	(0.32 - 3.23)	0.53	(0.10 - 2.83)
	Persons without cancer	187	42%	(28% - 57%)	53%	(45% - 62%)	25%	(16% - 35%)	72%	(62% - 80%)	0.48	(0.40 - 0.56)	0.83	(0.43 - 1.59)	0.88	(0.40 - 1.92)

Significant OR’s were indicated in bold.

*Abbreviations*: *QoL* Quality of Life, *aCGA* abbreviated Comprehensive Geriatric Assessment, *GFI* Groningen Frailty Indicator, *VES-13* Vulnerable Elders Survey-13, *Se* sensitivity, *Sp* specificity, *PPV* positive predictive value, *NPV* negative predictive value, *OR* odds ratio, *95% CI* 95% confidence interval.

^a^Functional decline was defined as deterioration one or more domains of ADL and/or IADL compared to baseline.

^b^Decline in QoL was defined as a difference of ≥ 10 points.

^c^Multivariate logistic regression: adjusted for age, gender, and number of medicines. For cancer patients we also adjusted for stage, type of cancer, and type of treatment.

#### QoL

Between baseline and one-year follow-up, 23% of older cancer patients and 30% of older persons without cancer experienced decline in QoL (*p* = 0.24). None of the geriatric screening tools was associated with a decline in QoL. Also for QoL, the predictive properties of all screening tools were poor with sensitivity ranging between and 40% and 67% (see Table 2). Specificity ranged between 37% and 54%. The positive predictive value ranged between 18% and 27% and the negative predictive value ranged between 67% and 80% for decline in QoL. The AUC ranged between 0.42 and 0.53 for decline in QoL.

### Sensitivity analyses

Results were robust for sensitivity analyses and did not change when mortality was included as endpoint for functional decline and decline in QoL (see Additional file 2: Table S2), nor when missing values for geriatric screening tools were imputed either as a worst- or best-case scenario (see Additional file 2: Table S3), when analyses were repeated for women only (see Additional file 2: Table S4), or when the operationalization of functional decline was altered (see Additional file 2: Table S5).

## Discussion

In geriatric oncology as well as in the primary care setting, geriatric screening tools are increasingly implemented in daily practice. However, firm evidence for their ability to predict relevant outcomes such as functional decline and decline in QoL is lacking. In the current study, we showed that the predictive properties of the geriatric screening tools were poor in a population of older (cancer) patients with a relatively good prognosis. Abnormal scores for the selected geriatric screening tools were associated with a higher risk of functional decline. However, this was only true in an older primary care population without a history of cancer, and only for univariate analyses. Furthermore, we found no evidence for increased risk for decline in QoL, and overall the sensitivity, specificity and predictive values of the geriatric screening tools were low.

### The diagnostic value of geriatric screening tools

The majority of studies on the usefulness of geriatric screening tools have focused on their ability to identify patients who might benefit from a full geriatric assessment. Hamaker et al. were the first to systematically review the diagnostic value of several geriatric screening tools against a full geriatric assessment in an oncogeriatric population [10]. They showed a median sensitivity of 51% for the aCGA, 57% for the GFI, and 68% for the VES-13. Only for the G8 sensitivity was high (87%). A recent update of the SIOG recommendations showed similar results [11]. Decoster et al. also showed that the sensitivity of the G8 was better compared to other tools, whereas results for the VES-13 were mixed. The superiority of the G8 compared to the VES-13 with respect to sensitivity was also confirmed by others [32,33]. For example, a recent large cohort study in France, showed a sensitivity of 77% for the G8 and 69% for the VES-13 [33]. We agree with the recommendations of Decoster et al.; “screening tools do not replace a full geriatric assessment but are recommended in a busy practice in order to identify those patients in need of a full geriatric assessment” [11].

In older persons without cancer similar results have been found as well. For example, a recent systematic review in community-dwelling older people showed a sensitivity of 58% for the GFI [34]. We showed a sensitivity of 74% for the GFI and 82% for the VES-13 in older persons without cancer [28].

It was however beyond the scope of the current study to investigate the diagnostic value of geriatric screening tools against a full geriatric assessment. In contrast, we aimed to assess the value of geriatric screening tools to predict decline in functional status and QoL in a population of older (cancer) patients with a good prognosis.

### The predictive value of geriatric screening tools

Recently, the predictive value of geriatric screening tools has received increasing attention. For example, a couple of larger studies have shown that abnormal scores for the G8 [13,33,35] and GFI [36] are predictive for mortality in a population of older cancer patients. However, data are still sparse and need confirmation by others (for a recent overview see Decoster et al. [11]).

#### Predicting functional decline in older cancer patients

To our knowledge, this is the first study to investigate the ability of the aCGA, GFI and VES-13 to predict functional decline in an oncogeriatric population, and only one study has investigated the predictive value of the G8 [13]. We found that in older cancer patients with a relatively good prognosis, neither the G8, nor the aCGA, GFI, or VES-13 seems to predict functional decline after one-year follow-up.

Kenis et al. studied the value of the G8 and the Flemish version of the Triage Risk Screening tool to predict functional decline after three months [13]. For the G8 and decline in ADL after three months, they showed a high sensitivity (84%) and a high negative predictive value (91%). In contrast, we showed a sensitivity of 64%, and a negative predictive value of 43% for the prediction of functional decline after one year of follow-up by the G8.

Our definition of functional impairment and functional decline was very inclusive, namely impairment or deterioration on at least one domain of ADL or IADL. However, when functional impairment was disentangled in impairment on ADL or IADL our results were similar to those reported by others. For example, we showed impairment in 51% and 56% of older cancer patients for ADL and IADL respectively. Similarly, Kenis et al. who measured functional status before start of treatment in a comparable population, showed impairment in 51% and 57% of older cancer patients for ADL and IADL respectively [13]. In order to test the influence of our definition of functional decline, we also calculated sensitivity, specificity, and positive and negative predictive values for decline in ADL and IADL separately. This did not change the interpretation of our results. For example, for predicting decline in ADL after one year of follow-up by the G8, sensitivity was 64% and negative predictive value 60%. It may be possible that geriatric screening tools are more suitable to predict outcomes such as functional decline on the short term. In this respect, a recent study showed that specific elements of a geriatric assessment, such as depression and impairment for IADL, predicted functional decline between the beginning of chemotherapy and the second cycle [37]. However, Huisman et al. showed no association between the GFI and VES-13 and 30-day morbidity after cancer surgery [38].

#### Predicting functional decline in an older primary care population

Compared to older cancer patients, the ability of geriatric screening tools to predict functional decline has been studied more extensively in a general older population without a history of cancer. We showed that abnormal scores for the selected geriatric screening tools were associated with a higher risk of functional decline in an older primary care population without a history of cancer. However, except for the VES-13, this was only true for univariate analyses and sensitivity was low ranging from 58% to 67% and an AUC between 0.60 and 0.64.

The screening tools used in a general older population are often different from those in an oncogeriatric setting. Nevertheless, Daniels et al. showed also for these tools, among which the GFI, that the predictive accuracy was poor with an area under the curve ranging between 0.54 and 0.67 [39]. From our selected screening tools, only the GFI and the VES-13 have previously been used in a general population as well. The G8 and aCGA have been developed especially for identifying older cancer patients who might benefit from an entire geriatric assessment. As such, the predictive validity of these tools has not been studied in persons without cancer.

For the GFI, Daniels et al. showed a significant association (sensitivity 71%, specificity 63%, and AUC 0.67) for predicting functional decline after one year of follow-up [39]. In our study, GFI was significantly associated with functional decline in univariate analyses only. Sensitivity in our study was lower (58%), but specificity and AUC were comparable (63% and 0.68 respectively). Other studies that evaluated the predictive value of the GFI were cross-sectional, used a different time frame or other operationalization of the outcome measurement. For example, Schuurmans et al. showed that the GFI was more strongly related to self-management abilities compared to age [40] and Peters et al. showed that GFI was significantly associated with ADL (data collected two weeks later) [41].

The VES-13 has been especially developed to identify community-dwelling vulnerable older people at risk for functional decline [31], and has been shown to be a good predictor of functional decline after one year, two years and even five years [31,42,43]. This was partly confirmed by our results; abnormal scores on the VES-13 were associated with functional decline in older persons without cancer, both in univariate and multivariate analyses. However, sensitivity, specificity and AUC were still low (62%, 66%, and 0.64 respectively).

#### Predicting decline in QoL

QoL is increasingly acknowledged as an important treatment goal, which is for example illustrated by the increasing number of studies that use QoL as a secondary endpoint [44]. However, to our knowledge, this is the first study that prospectively evaluates the ability of the aCGA, G8, GFI, and VES-13 to identify patients at risk for decline in QoL after one-year follow-up. We showed that abnormal scores on geriatric screening tools are not associated with decline in QoL, neither in older cancer patients, nor in persons without cancer.

In contrast to our results, previous studies have shown a negative association between mean Qol scores and abnormal scores on geriatric screening tools. A study among older cancer patients who underwent surgery showed that GFI scores were significantly associated with QoL at one and three months after surgery [45]. Although not based on a screening tool, but on a full geriatric assessment, Pottel et al. showed in a sample of older patients with head and neck cancer that vulnerable patients scored significantly lower on global QoL compared to fit patients [46]. In a sample of community-dwelling as well as institutionalized older persons, Peters et al. found a significant negative association between GFI score and QoL, which was measured two weeks later [41].

A possible explanation for our differing results is that we studied the association between the abnormal score on screening tools and decline in QoL instead of a mean QoL score. As QoL was already low at time of cancer diagnosis, only a small proportion of cancer patients deteriorated even further, which is probably due to a ceiling effect. In the current study, 23% of older cancer patients and 30% of persons without cancer experienced clinically relevant decline in QoL between baseline and one-year follow-up. Although not statistically significant, the proportion of cancer patients who experienced a relevant decline in QoL was smaller compared to persons without cancer. As expected, the mean QoL at baseline was significantly lower for older cancer patients compared to persons without cancer (p < 0.01). Similarly to our results, a Canadian study among cancer patients aged ≥65 years reported a decline in 23% of patients between baseline and one year follow-up [47]. A Danish study showed a decline in 30% of older cancer patients between cancer diagnosis and six months later [48].

### Strengths and limitations of this study

With exception of the G8, this is the first prospective study to evaluate the value of the aCGA, GFI, and VES-13 to predict functional decline and decline over a one-year time frame in QoL in older cancer patients. Furthermore, this is the first prospective study that evaluates the predictive value of geriatric screening tools in a population of older cancer patients and in an older primary care population without a history of cancer; the identification of persons at risk for functional decline or decline in QoL is relevant in both groups. This increases the generalizability of our results and shows that the predictive value of selected geriatric screening tools is poor in both groups. Another important strength of this study is the use of validated tools, which are commonly used in an oncogeriatric population [10].

Nevertheless, this study is also prone to some limitations. Although our selected tools are commonly used in an oncogeriatric population, these have been less extensively implemented in primary care. Furthermore, for cancer patients, it was not always possible to interview patients before start of treatment. However, we do not believe this influences our results as we showed that our results for functional status and QoL were similar to those of Kenis et al. and Puts et al. who did recruit patients before start of treatment [13,47]. Furthermore, the proportion of people with an abnormal score at baseline on the selected geriatric screening tools were comparable with those previously reported in cancer patients as well as a primary care population [11,41]. Another limitation is the relatively small sample size of cancer patients. For example we only had complete data on QoL for 67 older cancer patients. As such, it may be possible that the statistically non-significant results for older cancer patients were due to lack of power. Nevertheless, we showed that our results with respect to sensitivity and specificity were comparable to others and for QoL we showed that the operationalization of decline probably played a roll as well. Loss to follow-up and incomplete data is a frequent problem in studies among older patients. In our study, 23% of patients were lost to follow-up, 6% of patients skipped the data collection at one-year follow-up, and depending on the variable of interest between 0 and 22 persons had incomplete data. However, we do not believe that this affects the reliability of our results. First, patients who were excluded from analyses (due to loss to follow-up or skipping data collection) were comparable with persons included in the analyses with respect to baseline characteristics (see Additional file 1: Table S1). Except for the in- and excluded cancer patients, the distribution of cancer stage was different. Second, our results were robust for sensitivity analyses. Results did not change, neither when missing values for geriatric screening tools were imputed either as a worst- or best-case scenario, nor when mortality was included as endpoint for functional decline and decline in QoL (see Additional file 2: Table S2 and S3). Furthermore, the proportion lost to follow-up is comparable with other large health and ageing studies in Europe. For example 22% loss to follow-up in the English Longitudinal Study of Ageing (ELSA) [49] and 27% in the Survey of Health, Ageing and Retirement in Europe (SHARE) [50]. Moreover, similar to our study, the percentage of loss to follow-up remained the same when it was subdivided to persons who had been diagnosed with cancer and persons who had never been diagnosed with cancer [49].

## Conclusion

In this study we showed that the ability of the selected geriatric screening tools to identify patients at risk for functional decline or decline in QoL after one year is poor. This was the case for older cancer patients with a considerable life expectancy as well as older persons without cancer. Hence, a geriatric screening tool cannot be relied on in isolation, but they do provide very valuable information and may prompt physicians to also consider different aspects of functioning. These results need to be confirmed, as the number of prospective studies in an oncogeriatric population is still limited.

## Additional files

Additional file 1: Table S1. Baseline population characteristics for participants included and excluded from analyses. **Table S1.** shows the comparison between patients that were included and excluded from analysis.
Additional file 2: Table S2. Sensitivity analysis: mortality was included as functional decline or decline in QoL. **Table S3.** Sensitivity analysis: missing values were imputed as best and worst case scenario. **Table S4.** Sensitivity analysis: women separately. **Table S5.** Sensitivity analysis: predicting decline in ADL and IADL separately. **Tables S2 – S5.** present the sensitivity analyses. We repeated the analyses by including participants who died during one-year follow-up, by making a worst- and best-case scenario with imputing missing values as either a normal or abnormal score, by repeating the analyses for women separately, and by repeating the analyses for decline on ADL and IADL separately.

## Abbreviations

aCGA: Abbreviated comprehensive geriatric assessment
ADL: Activities of daily living
AUC: Area under the curve
EORTC QLQ-C30: European Organization for Research and Treatment of Cancer Quality of Life Questionnaire C30 version 3
GFI: Groningen Frailty Indicator
IADL: Instrumental activities of daily living
VES-13: Vulnerable Elders Survey
QoL: Quality of life
