# Supplementary material for: Geriatric screening tools are of limited value to predict decline in functional status and quality of life: results of a cohort study
Source: BMC Fam Pract. 2015 Mar 3;16:30. doi: 10.1186/s12875-015-0241-x (PMC4358725; doi:10.1186/s12875-015-0241-x)
Supplement: Additional file 1: Table S1. — Baseline population characteristics for participants included and excluded from analyses. Table S1. shows the comparison between patients that were included and excluded from analysis. [file 12875_2015_241_MOESM1_ESM.pdf]

Additional Table 1. Baseline population characteristics for participants included and excluded from analyses[a]

	Older cancer patients					Older persons without cancer				
	Analysed N = 134		Excluded N = 46			Analysed N = 220		Excluded N = 103		
	N	%	N	%	P-value	N	%	N	%	P-value
**Age: mean (SD)**	77.1	(5.11)	77.46	(4.86)	0.68	78.13	(5.47)	78.26	(5.60)	0.84
**Gender**					0.40					0.69
Male	28	21%	7	15%		82	37%	36	35%	
Female	106	79%	39	85%		138	63%	67	65%	
**Living conditions**					0.83					0.51
Alone	44	33%	12	26%		72	33%	27	26%	
With partner	71	53%	26	57%		124	56%	62	60%	
With friends/family	11	8%	5	11%		20	9%	13	13%	
Institutionalised	8	6%	3	7%		4	2%	1	1%	
**Cancer site**					0.98					
Breast	93	69%	32	70%						
Colorectal	41	31%	14	30%						
**Cancer treatment**										
Surgery	125	93%	41	89%	0.28					
Chemotherapy	39	29%	19	41%	0.13					
Radiotherapy	70	52%	27	59%	0.45					
Hormonal therapy	53	40%	17	37%	0.75					
Immunotherapy	19	14%	3	7%	0.17					
Missing	3	2%	1	2%						
**Cancer Stage**					**0.04**					
I	22	16%	10	22%						
II	72	54%	17	37%						
III	18	13%	13	28%						
Missing	22	16%	6	13%						
**Geriatric screening tools**										
aCGA: Abnormal score	80	70%	27	59%	0.42	118	58%	67	68%	0.11
G8: Abnormal score	79	64%	22	51%	0.13	114	54%	54	56%	0.72
GFI: Abnormal score	36	49%	11	48%	0.95	94	47%	54	57%	0.08
VES-13: Abnormal score	43	51%	19	61%	0.31	104	49%	62	61%	0.05
**Functional impairment**	97	72%	30	65%	0.36	141	65%	69	67%	0.61
**QoL: mean (SD)** [b]	69.93	(19.30)	67.5	(23.20)	0.57	75.91	(17.84)	72.39	(19.41)	0.11

Abbreviations: QoL, Quality of Life [a] Patients excluded from analyses were patients lost to follow-up, who skipped one year follow-up data collection, or who received a diagnosis of cancer (only applicable to persons without cancer); [b] In one hospital, data collection was integrated in a routine geriatric assessment, therefore QoL was not assessed in the 35 older cancer patients recruited in this hospital.
