# Supplementary material for: Geriatric screening tools are of limited value to predict decline in functional status and quality of life: results of a cohort study
Source: BMC Fam Pract. 2015 Mar 3;16:30. doi: 10.1186/s12875-015-0241-x (PMC4358725; doi:10.1186/s12875-015-0241-x)
Supplement: Additional file 2: Table S2. — Sensitivity analysis: mortality was included as functional decline or decline in QoL. Table S3. Sensitivity analysis: missing values were imputed as best and worst case scenario. Table S4. Sensitivity analysis: women separately. Table S5. Sensitivity analysis: predicting decline in ADL and IADL separately. Tables S2 – S5. present the sensitivity analyses. We repeated the analyses by including participants who died during one-year follow-up, by making a worst- and best-case scenario with imputing missing values as either a normal or abnormal score, by repeating the analyses for women separately, and by repeating the analyses for decline on ADL and IADL separately. [file 12875_2015_241_MOESM2_ESM.pdf]

Additional Table 2. Sensitivity analysis: mortality was included as functional decline or decline in QoL[a]

			Sensitivity		Specificity		Positive predictive value		Negative predictive value		Area under the curve		Univariate logistic regression		Multivariate logistic regression[d]	
		**N total**	**Se**	**(95% CI)**	**Sp**	**(95% CI)**	**PPV**	**(95% CI)**	**NPV**	**(95% CI)**	**AUC**	**(95% CI)**	**OR**	**(95% CI)**	**OR**	**(95% CI)**
**Functional decline[b]**																
**aCGA**	Cancer patients	122	73%	(61% - 82%)	33%	(20% - 48%)	62%	(51% - 72%)	44%	(28% - 62%)	0.53	(0.44 - 0.61)	1.28	(0.58 - 2.83)	0.80	(0.30 - 2.12)
	Persons without cancer	191	69%	(57% - 79%)	52%	(43% - 62%)	51%	(41% - 61%)	70%	(59% - 80%)	0.61	(0.54 - 0.67)	**2.41**	**(1.32 - 4.39)**	1.90	(0.94 - 3.86)
**G8**	Cancer patients	122	63%	(51% - 74%)	37%	(23% - 52%)	60%	(48% - 71%)	40%	(26% - 56%)	0.50	(0.41 - 0.59)	0.99	(0.47 - 2.10)	0.60	(0.24 - 1.49)
	Persons without cancer	191	66%	(55% - 76%)	57%	(47% - 66%)	53%	(42% - 63%)	70%	(59% - 79%)	0.62	(0.55 - 0.69)	**2.58**	**(1.42 - 4.68)**	1.83	(0.91 - 3.65)
**GFI**	Cancer patients	74	60%	(43% - 74%)	59%	(41% - 76%)	66%	(49% - 80%)	53%	(36% - 70%)	0.59	(0.48 - 0.71)	2.15	(0.84 - 5.48)	1.47	(0.44 - 4.92)
	Persons without cancer	191	60%	(48% - 71%)	63%	(53% - 72%)	54%	(43% - 65%)	69%	(59% - 78%)	0.62	(0.55 - 0.69)	**2.56**	**(1.42 - 4.62)**	1.85	(0.93 - 3.70)
**VES-13**	Cancer patients	74	57%	(41% - 72%)	66%	(47% - 81%)	69%	(51% - 83%)	54%	(37% - 70%)	0.61	(0.50 - 0.73)	2.55	(0.98 - 6.59)	2.68	(0.68 - 10.54)
	Persons without cancer	191	64%	(52% - 74%)	66%	(56% - 75%)	57%	(46% - 68%)	72%	(62% - 80%)	0.65	(0.58 - 0.72)	**3.38**	**(1.85 - 6.16)**	**2.89**	**(1.37 - 6.07)**
**Decline in Qol[c]**																
**aCGA**	Cancer patients	74	59%	(36% - 79%)	37%	(24% - 51%)	28%	(16% - 44%)	68%	(48% - 84%)	0.48	(0.35 - 0.60)	0.83	(0.30 - 2.31)	0.86	(0.26 - 2.83)
	Persons without cancer	191	59%	(45% - 72%)	45%	(36% - 53%)	30%	(21% - 39%)	74%	(63% - 83%)	0.52	(0.44 - 0.60)	1.17	(0.62 - 2.21)	1.17	(0.57 - 2.42)
**G8**	Cancer patients	74	64%	(41% - 83%)	39%	(25% - 53%)	30%	(18% - 46%)	71%	(51% - 87%)	0.51	(0.39 - 0.63)	1.09	(0.39 - 3.07)	1.02	(0.31 - 3.36)
	Persons without cancer	191	44%	(31% - 59%)	44%	(35% - 53%)	24%	(16% - 33%)	67%	(56% - 76%)	0.44	(0.36 - 0.52)	0.62	(0.33 - 1.18)	0.50	(0.24 - 1.06)
**GFI**	Cancer patients	74	55%	(32% - 76%)	50%	(36% - 64%)	32%	(18% - 49%)	72%	(55% - 86%)	0.52	(0.40 - 0.65)	1.20	(0.44 - 3.26)	1.27	(0.38 - 4.20)
	Persons without cancer	191	46%	(33% - 60%)	53%	(45% - 62%)	28%	(19% - 39%)	72%	(62% - 80%)	0.50	(0.42 - 0.58)	0.98	(0.52 - 1.85)	0.91	(0.44 - 1.89)
**VES-13**	Cancer patients	74	50%	(28% - 72%)	54%	(40% - 68%)	31%	(17% - 49%)	72%	(55% - 85%)	0.52	(0.39 - 0.65)	1.17	(0.43 - 3.16)	1.07	(0.28 - 4.16)
	Persons without cancer	191	46%	(33% - 60%)	53%	(45% - 62%)	28%	(19% - 39%)	72%	(62% - 80%)	0.50	(0.42 - 0.58)	0.98	(0.52 - 1.85)	0.91	(0.43 - 1.96)

Abbreviations: QoL, Quality of Life; aCGA, abbreviated Comprehensive Geriatric Assessment; GFI, Groningen Frailty Indicator; VES-13, Vulnerable Elders Survey-13; Se, sensitivity; Sp, specificity; PPV, positive predictive value; NPV, negative predictive value, OR, odds ratio; 95% CI, 95% confidence interval; [a] For the diagnostic accuracy of geriatric screening tools to predict decline in functional status and QoL, mortality, defined as death within one year after baseline evaluation, was included as a positive endpoint, for both functional decline or decline in QoL; [b] Functional decline was defined as deterioration one or more domains of ADL and/or IADL compared to baseline; [c] Decline in QoL was defined as a difference of ≥ 10 points; [d] Multivariate logistic regression: adjusted for age, gender, and number of medicines. For cancer patients we also adjusted for stage, type of cancer, and type of treatment.

Additional Table 3. Sensitivity analysis: missing values were imputed as best and worst case scenario[a]

			Sensitivity		Specificity		Positive predictive value		Negative predictive value		Area under the curve		Univariate logistic regression		Multivariate logistic regression[d]	
		N total	Se	(95% CI)	Sp	(95% CI)	PPV	(95% CI)	NPV	(95% CI)	AUC	(95% CI)	OR	(95% CI)	OR	(95% CI)
**Best case scenario**																
**Functional decline[b]**																
**aCGA**	Cancer patients	125	64%	(52% - 75%)	46%	(32% - 61%)	63%	(51% - 74%)	48%	(34% - 63%)	0.55	(0.47 - 0.64)	1.55	(0.75 - 3.20)	1.05	(0.45 - 2.49)
	Persons without cancer	212	60%	(50% - 71%)	53%	(44% - 62%)	49%	(40% - 59%)	64%	(54% - 73%)	0.57	(0.50 - 0.63)	**1.72**	**(0.99 - 2.98)**	1.44	(0.77 - 2.71)
**G8**	Cancer patients	125	63%	(51% - 74%)	37%	(24% - 51%)	58%	(47% - 69%)	41%	(27% - 57%)	0.50	(0.41 - 0.58)	0.98	(0.47 - 2.05)	0.60	(0.25 - 1.44)
	Persons without cancer	212	64%	(53% - 74%)	55%	(46% - 64%)	52%	(42% - 61%)	67%	(57% - 76%)	0.60	(0.53 - 0.66)	**2.18**	**(1.25 - 3.81)**	**1.75**	(0.91 - 3.33)
**GFI**	Cancer patients	83	50%	(35% - 65%)	64%	(47% - 79%)	61%	(44% - 77%)	53%	(38% - 68%)	0.57	(0.46 - 0.68)	1.79	(0.74 - 4.31)	1.47	(0.49 - 4.42)
	Persons without cancer	212	55%	(44% - 65%)	65%	(55% - 73%)	54%	(43% - 64%)	66%	(56% - 74%)	0.60	(0.53 - 0.66)	**2.21**	**(1.27 - 3.86)**	**2.00**	**(1.07 - 3.74)**
**VES-13**	Cancer patients	83	64%	(48% - 78%)	64%	(47% - 79%)	67%	(51% - 80%)	61%	(45% - 76%)	0.64	(0.53 - 0.74)	**3.13**	**(1.27 - 7.67)**	3.16	(0.87 - 11.53)
	Persons without cancer	212	63%	(52% - 73%)	63%	(54% - 71%)	56%	(46% - 66%)	69%	(60% - 78%)	0.63	(0.56 - 0.69)	**2.83**	**(1.61 - 4.97)**	**2.85**	**(1.45 - 5.59)**
**Decline in Qol[c]**																
**aCGA**	Cancer patients	83	47%	(24% - 71%)	48%	(36% - 61%)	21%	(10% - 37%)	76%	(60% - 88%)	0.48	(0.35 - 0.61)	0.85	(0.30 - 2.36)	0.80	(0.24 - 2.67)
	Persons without cancer	212	56%	(43% - 68%)	48%	(40% - 57%)	31%	(23% - 41%)	72%	(62% - 81%)	0.52	(0.45 - 0.59)	1.17	(0.65 - 2.11)	1.32	(0.68 - 2.58)
**G8**	Cancer patients	83	74%	(49% - 91%)	34%	(23% - 47%)	25%	(14% - 38%)	82%	(62% - 94%)	0.54	(0.42 - 0.66)	1.47	(0.47 - 4.60)	1.43	(0.39 - 5.29)
	Persons without cancer	212	44%	(32% - 58%)	44%	(36% - 52%)	25%	(17% - 34%)	65%	(55% - 74%)	0.44	(0.37 - 0.51)	0.62	(0.34 - 1.12)	0.55	(0.27 - 1.10)
**GFI**	Cancer patients	83	37%	(16% - 62%)	55%	(42% - 67%)	19%	(8% - 36%)	75%	(60% - 86%)	0.46	(0.33 - 0.59)	0.70	(0.25 - 2.02)	0.58	(0.16 - 2.08)
	Persons without cancer	212	41%	(29% - 54%)	55%	(47% - 63%)	28%	(19% - 38%)	69%	(60% - 77%)	0.48	(0.41 - 0.56)	0.86	(0.47 - 1.56)	0.94	(0.49 - 1.83)
**VES-13**	Cancer patients	83	53%	(29% - 76%)	50%	(37% - 63%)	24%	(12% - 40%)	78%	(62% - 89%)	0.51	(0.38 - 0.64)	1.11	(0.40 - 3.10)	0.54	(0.12 - 2.51)
	Persons without cancer	212	46%	(33% - 59%)	51%	(43% - 59%)	28%	(20% - 38%)	69%	(60% - 78%)	0.49	(0.41 - 0.56)	0.89	(0.49 - 1.60)	0.94	(0.47 - 1.88)
**Worst case scenario**																
**Functional decline[a]**																
**aCGA**	Cancer patients	125	75%	(64% - 85%)	31%	(19% - 45%)	60%	(50% - 71%)	47%	(30% - 65%)	0.53	(0.45 - 0.61)	1.36	(0.61 - 3.00)	0.88	(0.34 - 2.30)
	Persons without cancer	212	73%	(62% - 81%)	50%	(40% - 59%)	52%	(43% - 61%)	71%	(60% - 80%)	0.61	(0.55 - 0.68)	**2.60**	**(1.45 - 4.65)**	**2.57**	**(1.32 - 5.00)**
**G8**	Cancer patients	125	66%	(54% - 77%)	37%	(24% - 51%)	59%	(48% - 70%)	43%	(28% - 59%)	0.51	(0.43 - 0.60)	1.11	(0.53 - 2.32)	0.67	(0.28 - 1.63)
	Persons without cancer	212	65%	(54% - 75%)	54%	(44% - 63%)	51%	(42% - 61%)	67%	(57% - 76%)	0.59	(0.53 - 0.66)	**2.14**	**(1.22 - 3.74)**	**1.69**	(0.88 - 3.25)

		N total	Sensitivity Se	(95% CI)	Specificity Sp	(95% CI)	Positive predictive value PPV	(95% CI)	Negative predictive value NPV	(95% CI)	Area under the curve AUC	(95% CI)	Univariate logistic regression OR	(95% CI)	Multivariate logistic regression[d] OR	(95% CI)
**GFI**	Cancer patients	83	61%	(46% - 76%)	51%	(35% - 68%)	59%	(43% - 73%)	54%	(37% - 71%)	0.56	(0.46 - 0.67)	1.67	(0.70 - 4.00)	0.96	(0.30 - 3.03)
	Persons without cancer	212	59%	(49% - 70%)	59%	(49% - 68%)	52%	(42% - 62%)	66%	(56% - 75%)	0.59	(0.52 - 0.66)	**2.07**	**(1.19 - 3.60)**	1.70	(0.89 - 3.24)
**VES-13**	Cancer patients	83	64%	(48% - 78%)	62%	(45% - 77%)	65%	(49% - 79%)	60%	(43% - 75%)	0.63	(0.52 - 0.73)	**2.80**	**(1.15 - 6.82)**	2.85	(0.81 - 10.04)
	Persons without cancer	212	65%	(54% - 75%)	62%	(53% - 71%)	56%	(46% - 66%)	70%	(61% - 79%)	0.63	(0.57 - 0.70)	**3.01**	**(1.71 - 5.29)**	**3.12**	**(1.57 - 6.20)**
**Decline in Qol[c]**																
**aCGA**	Cancer patients	83	58%	(34% - 80%)	31%	(20% - 44%)	20%	(10% - 33%)	71%	(51% - 87%)	0.45	(0.32 - 0.57)	0.63	(0.22 - 1.79)	0.46	(0.11 - 1.88)
	Persons without cancer	212	65%	(52% - 77%)	42%	(34% - 51%)	32%	(24% - 41%)	74%	(64% - 83%)	0.54	(0.47 - 0.61)	1.37	(0.74 - 2.52)	1.60	(0.81 - 3.18)
**G8**	Cancer patients	83	74%	(49% - 91%)	34%	(23% - 47%)	25%	(14% - 38%)	82%	(62% - 94%)	0.54	(0.42 - 0.66)	1.47	(0.47 - 4.60)	1.43	(0.39 - 5.29)
	Persons without cancer	212	46%	(33% - 59%)	42%	(34% - 51%)	25%	(18% - 34%)	65%	(55% - 74%)	0.44	(0.37 - 0.52)	0.62	(0.35 - 1.13)	0.55	(0.27 - 1.12)
**GFI**	Cancer patients	83	47%	(24% - 71%)	42%	(30% - 55%)	20%	(9% - 34%)	73%	(56% - 86%)	0.45	(0.32 - 0.58)	0.66	(0.23 - 1.84)	0.39	(0.10 - 1.54)
	Persons without cancer	212	49%	(36% - 62%)	51%	(43% - 59%)	30%	(21% - 40%)	70%	(61% - 79%)	0.50	(0.43 - 0.58)	1.01	(0.56 - 1.82)	1.16	(0.58 - 2.31)
**VES-13**	Cancer patients	83	58%	(34% - 80%)	50%	(37% - 63%)	26%	(14% - 41%)	80%	(64% - 91%)	0.54	(0.41 - 0.67)	1.38	(0.49 - 3.87)	0.79	(0.18 - 3.46)
	Persons without cancer	212	49%	(36% - 62%)	50%	(42% - 59%)	30%	(21% - 39%)	70%	(61% - 79%)	0.50	(0.42 - 0.57)	0.98	(0.54 - 1.77)	1.08	(0.54 - 2.16)

Abbreviations: QoL, Quality of Life; aCGA, abbreviated Comprehensive Geriatric Assessment; GFI, Groningen Frailty Indicator; VES-13, Vulnerable Elders Survey-13; Se, sensitivity; Sp, specificity; PPV, positive predictive value; NPV, negative predictive value, OR, odds ratio; 95% CI, 95% confidence interval

[a] For the diagnostic accuracy of geriatric screening tools to predict decline in functional status and QoL, we performed a sensitivity analysis to assess the influence of missing values, making a worst- and best-case scenario by imputing missing values as either a normal or abnormal score; [b] Functional decline was defined as deterioration one or more domains of ADL and/or IADL compared to baseline; [c] Decline in QoL was defined as a difference of ≥ 10 points; [d] Multivariate logistic regression: adjusted for age, gender, and number of medicines. For cancer patients we also adjusted for stage, type of cancer, and type of treatment.

Additional Table 4. Sensitivity analysis: women separately[a]

			Sensitivity		Specificity		Positive predictive value		Negative predictive value		Area under the curve		Univariate logistic regression		Multivariate logistic regression[d]	
		N total	Se	(95% CI)	Sp	(95% CI)	PPV	(95% CI)	NPV	(95% CI)	AUC	(95% CI)	OR	(95% CI)	OR	(95% CI)
**Functional decline[b]**																
**aCGA**	Cancer patients	97	75%	(62% - 86%)	33%	(19% - 49%)	61%	(49% - 73%)	48%	(29% - 68%)	0.54	(0.45 - 0.63)	1.48	(0.60 - 3.62)	2.30	(1.01 - 5.22)
	Persons without cancer	120	74%	(59% - 87%)	44%	(33% - 56%)	43%	(31% - 55%)	76%	(61% - 87%)	0.59	(0.51 - 0.68)	0.80	(0.27 - 2.33)	1.84	(0.75 - 4.54)
**G8**	Cancer patients	97	60%	(46% - 72%)	38%	(23% - 54%)	58%	(44% - 70%)	40%	(24% - 57%)	0.49	(0.39 - 0.59)	0.89	(0.39 - 2.04)	**3.08**	**(1.39 - 6.79)**
	Persons without cancer	120	70%	(54% - 83%)	57%	(45% - 68%)	48%	(35% - 61%)	77%	(64% - 87%)	0.64	(0.55 - 0.72)	0.54	(0.20 - 1.46)	**2.80**	**(1.13 - 6.95)**
**GFI**	Cancer patients	50	59%	(39% - 78%)	57%	(35% - 77%)	62%	(41% - 80%)	54%	(33% - 74%)	0.58	(0.44 - 0.72)	1.89	(0.61 - 5.83)	1.14	(0.27 - 4.74)
	Persons without cancer	120	61%	(44% - 75%)	56%	(44% - 67%)	43%	(31% - 57%)	72%	(59% - 83%)	0.58	(0.49 - 0.67)	1.93	(0.91 - 4.13)	1.52	(0.64 - 3.60)
**VES-13**	Cancer patients	50	67%	(46% - 84%)	57%	(35% - 77%)	64%	(44% - 81%)	59%	(36% - 79%)	0.62	(0.48 - 0.75)	2.60	(0.82 - 8.20)	2.25	(0.51 - 10.03)
	Persons without cancer	120	72%	(56% - 85%)	60%	(48% - 71%)	50%	(37% - 63%)	79%	(67% - 89%)	0.66	(0.57 - 0.75)	**3.83**	**(1.71 - 8.59)**	**3.56**	**(1.44 - 8.81)**
**Decline in Qol[c]**																
**aCGA**	Cancer patients	50	54%	(25% - 81%)	35%	(20% - 53%)	23%	(10% - 41%)	68%	(43% - 87%)	0.45	(0.28 - 0.61)	0.63	(0.18 - 2.28)	0.47	(0.09 - 2.34)
	Persons without cancer	120	67%	(48% - 82%)	39%	(29% - 50%)	29%	(19% - 41%)	76%	(61% - 87%)	0.53	(0.43 - 0.63)	1.28	(0.55 - 2.98)	1.46	(0.57 - 3.71)
**G8**	Cancer patients	50	62%	(32% - 86%)	46%	(30% - 63%)	29%	(13% - 49%)	77%	(55% - 92%)	0.54	(0.38 - 0.70)	1.36	(0.37 - 4.95)	1.25	(0.29 - 5.29)
	Persons without cancer	120	42%	(26% - 61%)	44%	(33% - 55%)	22%	(13% - 35%)	67%	(53% - 79%)	0.43	(0.33 - 0.53)	0.57	(0.25 - 1.28)	0.47	(0.18 - 1.25)
**GFI**	Cancer patients	50	46%	(19% - 75%)	46%	(30% - 63%)	23%	(9% - 44%)	71%	(49% - 87%)	0.46	(0.30 - 0.62)	0.73	(0.21 - 2.59)	0.62	(0.14 - 2.77)
	Persons without cancer	120	42%	(26% - 61%)	47%	(36% - 58%)	23%	(13% - 36%)	68%	(55% - 80%)	0.45	(0.35 - 0.55)	0.66	(0.29 - 1.47)	0.66	(0.26 - 1.66)
**VES-13**	Cancer patients	50	54%	(25% - 81%)	43%	(27% - 61%)	25%	(11% - 45%)	73%	(50% - 89%)	0.49	(0.32 - 0.65)	0.89	(0.25 - 3.16)	0.44	(0.08 - 2.47)
	Persons without cancer	120	49%	(31% - 67%)	47%	(36% - 58%)	26%	(16% - 39%)	71%	(57% - 82%)	0.48	(0.38 - 0.58)	0.84	(0.38 - 1.87)	0.86	(0.34 - 2.16)

Abbreviations: QoL, Quality of Life; aCGA, abbreviated Comprehensive Geriatric Assessment; GFI, Groningen Frailty Indicator; VES-13, Vulnerable Elders Survey-13; Se, sensitivity; Sp, specificity; PPV, positive predictive value; NPV, negative predictive value, OR, odds ratio; 95% CI, 95% confidence interval; [a] The diagnostic accuracy of geriatric screening tools to predict decline in functional status and QoL in women separately, because the proportion of women in the group of cancer patients was higher as compared to the primary care population without cancer due to the inclusion of breast cancer patients; [b] Functional decline was defined as deterioration one or more domains of ADL and/or IADL compared to baseline; [c] Decline in QoL was defined as a difference of ≥ 10 points; [d] Multivariate logistic regression: adjusted for age, gender, and number of medicines. For cancer patients we also adjusted for stage, type of cancer, and type of treatment.

Additional Table 5. Sensitivity analysis: predicting decline in ADL and IADL separately[a]

			Sensitivity		Specificity		Positive predictive value		Negative predictive value		Area under the curve		Univariate logistic regression		Multivariate logistic regression[d]	
		**N total**	**Se**	**(95% CI)**	**Sp**	**(95% CI)**	**PPV**	**(95% CI)**	**NPV**	**(95% CI)**	**AUC**	**(95% CI)**	**OR**	**(95% CI)**	**OR**	**(95% CI)**
**Decline in ADL[b]**																
**aCGA**	Cancer patients	111	71%	(55% - 83%)	33%	(22% - 45%)	41%	(30% - 53%)	63%	(45% - 79%)	0.52	(0.43 - 0.61)	1.17	(0.51 - 2.66)	0.49	(0.17 - 1.42)
	Persons without cancer	186	60%	(42% - 75%)	46%	(38% - 54%)	21%	(14% - 31%)	82%	(72% - 90%)	0.53	(0.44 - 0.62)	1.23	(0.59 - 2.56)	0.59	(0.25 - 1.41)
**G8**	Cancer patients	111	64%	(48% - 78%)	36%	(25% - 49%)	39%	(28% - 52%)	60%	(43% - 75%)	0.50	(0.41 - 0.59)	0.98	(0.44 - 2.16)	0.47	(0.17 - 1.29)
	Persons without cancer	186	65%	(48% - 80%)	51%	(43% - 59%)	25%	(17% - 35%)	85%	(76% - 92%)	0.58	(0.49 - 0.67)	1.92	(0.91 - 4.06)	1.30	(0.54 - 3.17)
**GFI**	Cancer patients	64	55%	(32% - 76%)	55%	(39% - 70%)	39%	(22% - 58%)	70%	(51% - 84%)	0.55	(0.42 - 0.68)	1.45	(0.52 - 4.09)	0.61	(0.15 - 2.48)
	Persons without cancer	186	65%	(48% - 80%)	60%	(51% - 68%)	29%	(19% - 40%)	87%	(79% - 93%)	0.62	(0.54 - 0.71)	**2.74**	**(1.29 - 5.80)**	1.80	(0.77 - 4.21)
**VES-13**	Cancer patients	64	55%	(32% - 76%)	57%	(41% - 72%)	40%	(23% - 59%)	71%	(53% - 85%)	0.56	(0.43 - 0.69)	1.60	(0.57 - 4.52)	0.58	(0.12 - 2.72)
	Persons without cancer	186	62%	(45% - 78%)	59%	(51% - 67%)	27%	(18% - 38%)	86%	(78% - 92%)	0.61	(0.52 - 0.70)	**2.37**	**(1.13 - 4.97)**	**1.39**	**(0.58 - 3.33)**
**Decline in IADL[c]**																
**aCGA**	Cancer patients	111	82%	(67% - 93%)	36%	(25% - 48%)	41%	(30% - 53%)	79%	(61% - 91%)	0.59	(0.51 - 0.67)	2.58	(1.00 - 6.67)	2.46	(0.75 - 8.10)
	Persons without cancer	182	78%	(64% - 89%)	53%	(44% - 62%)	36%	(27% - 46%)	88%	(79% - 94%)	0.66	(0.58 - 0.73)	**4.05**	**(1.86 - 8.81)**	**6.25**	**(2.46 - 15.92)**
**G8**	Cancer patients	111	64%	(47% - 79%)	38%	(26% - 50%)	36%	(25% - 48%)	66%	(49% - 80%)	0.51	(0.41 - 0.60)	1.07	(0.48 - 2.41)	0.91	(0.34 - 2.45)
	Persons without cancer	182	67%	(52% - 81%)	54%	(45% - 62%)	33%	(24% - 43%)	83%	(73% - 90%)	0.61	(0.53 - 0.69)	**2.39**	**(1.19 - 4.83)**	2.25	(0.99 - 5.11)
**GFI**	Cancer patients	63	58%	(34% - 80%)	55%	(39% - 70%)	36%	(19% - 55%)	75%	(57% - 89%)	0.56	(0.43 - 0.70)	1.65	(0.56 - 4.89)	3.80	(0.75 - 19.19)
	Persons without cancer	182	57%	(41% - 71%)	59%	(50% - 67%)	32%	(22% - 43%)	80%	(71% - 87%)	0.58	(0.49 - 0.66)	1.86	(0.95 - 3.65)	1.87	(0.84 - 4.16)
**VES-13**	Cancer patients	63	63%	(38% - 84%)	61%	(46% - 76%)	41%	(24% - 61%)	79%	(62% - 91%)	0.62	(0.49 - 0.76)	2.72	(0.90 - 8.28)	**7.98**	**(1.19 - 53.64)**
	Persons without cancer	182	67%	(52% - 81%)	63%	(55% - 71%)	38%	(28% - 50%)	85%	(77% - 91%)	0.65	(0.57 - 0.73)	**3.55**	**(1.75 - 7.22)**	**5.87**	**(2.30 - 14.97)**

Abbreviations: ADL, activities of daily living; IADL, instrumental activities of daily living; aCGA, abbreviated Comprehensive Geriatric Assessment; GFI, Groningen Frailty Indicator; VES-13, Vulnerable Elders Survey-13; QoL, Quality of Life; Se, sensitivity; Sp, specificity; PPV, positive predictive value; NPV, negative predictive value, OR, odds ratio; 95% CI, 95% confidence interval; [a] The diagnostic accuracy of geriatric screening tools to predict decline in ADL and IALD separately; [b] Decline in ADL was defined as deterioration on one or more domains of ADL compared to baseline; [c] Decline in IADL was defined as deterioration on one or more domains of ADL compared to baseline; [d] Multivariate logistic regression: adjusted for age, gender, and number of medicines. For cancer patients we also adjusted for stage, type of cancer, and type of treatment.
